# Barriers to physical activity among adults in primary healthcare units in the National Health System: a cross-sectional study in Brazil

**DOI:** 10.1590/1516-3180.2021.0757.R1.20122021

**Published:** 2022-08-29

**Authors:** Ana Luísa Kuehn de Souza, Letícia Pechnicki dos Santos, Cassiano Ricardo Rech, Ciro Romelio Rodriguez-Añez, Claudia Alberico, Lucélia Justino Borges, Rogério César Fermino

**Affiliations:** I BHSc. Student, Multiprofessional Residency Program on Family Health, Sergio Arouca National School of Public Health, Fundação Oswaldo Cruz (FIOCRUZ), Rio de Janeiro (RJ), Brazil.; II MSc. Researcher, Postgraduate Program on Physical Education and Research Group on Environment, Physical Activity and Health, Universidade Tecnológica Federal do Paraná (UTFPR), Curitiba (PR), Brazil.; III PhD. Professor and Researcher, Postgraduate Program on Physical Education and Study Group on Urban Environment and Health, Universidade Federal de Santa Catarina (UFSC), Florianópolis (SC), Brazil.; IV PhD. Professor and Researcher, Postgraduate Program on Physical Education and Research Group on Environment, Physical Activity and Health, Universidade Tecnológica Federal do Paraná (UTFPR), Curitiba (PR), Brazil.; V PhD. Researcher, Biomedical/Biotechnology Research Institute, North Carolina Central University (NCCU), Durham (NC), United States.; VI PhD. Professor and Researcher, Department of Physical Education, Universidade Federal do Paraná (UFPR), Curitiba (PR), Brazil.; VII PhD. Professor and Researcher, Postgraduate Program on Physical Education and Research Group on Environment, Physical Activity and Health, Universidade Tecnológica Federal do Paraná (UTFPR), Curitiba (PR), Brazil.

**Keywords:** Motor activity, Exercise, Counseling, Primary health care, Public health, Epidemiologic studies, Leisure activities, Lifestyle, Intrapersonal barriers, Health care access barriers, Brazilian National Health System, SUS

## Abstract

**BACKGROUND:**

Many factors may negatively impact physical activity (PA), but studies lack evidence of individual predictors of perceived barriers to PA among adults in primary healthcare units.

**OBJECTIVE:**

To analyze associations between sociodemographic characteristics, health conditions, leisure-time physical activity (LTPA), PA counseling and perceived barriers to LTPA among adult patients in primary healthcare units of the National Health System in Brazil.

**DESIGN AND SETTING:**

Cross-sectional study on a representative sample of adults in primary healthcare units in São José dos Pinhais, Paraná, Brazil.

**METHODS:**

This study was conducted in 2019, among 779 adults (70% women). Barriers to LTPA, sociodemographic characteristics (sex, age, marital status, skin color, education and income), health conditions (body mass index, hypertension, diabetes, dyslipidemia, coronary disease and medications), LTPA level and PA counseling received were measured using validated, standardized procedures. The data were analyzed using chi-square and Mann-Whitney U tests.

**RESULTS:**

The most prevalent barriers were “feeling too tired” (53%) and “lack of time” (52%). PA counseling was inversely associated with “lack of time” (45% versus 57%; P < 0.001) but positively associated with “injury or disease” (38% versus 29%; P = 0.008). There was an inverse linear trend between the number of barriers and LTPA (walking and total) (P < 0.001). Most barriers differed in comparisons of sociodemographic characteristics, health conditions, LTPA and counseling (P < 0.05).

**CONCLUSIONS:**

The barriers vary according to the individual predictors. Counseling strategies need to be specific for each barrier and may be promising for promoting LTPA within primary healthcare.

## INTRODUCTION

Around 28% of the worldwide adult population is not physically active, with higher prevalence among women (32%) and Latin American populations (39%), especially in Brazil (47%).^1^

Therefore, physical inactivity has been considered to be a pandemic, with significant impact on public health and elevated economic burden with regard to healthcare, lack of productivity and premature mortality rates due to chronic disease.^2^ In Brazil, the National Policy on Health Promotion is an important strategy for confronting physical inactivity that guides physical activity promotion at the community level through primary healthcare.^3^

There is strong evidence showing the protective effects of regular physical activity against major chronic diseases and for improvement of quality of life.^2,4^ Leisure-time physical activity (LTPA) has higher potential for modification than physical activity at work, for example. Additionally, it is affected by psychological, biological, social and behavioral processes that operate at individual, group and social levels.^5^ Many factors can affect choices and opportunities for being physically active during leisure time,^6,7^ which demonstrates that LTPA is influenced by factors that go beyond motivation or the population's knowledge of its health benefits.^2^ Therefore, the perception of barriers to LTPA needs to be considered with regard to the success of programs to promote physical activity,^8^ since those barriers can differ according to sex, age, health conditions, social and cultural characteristics, types of LTPA and lifestyle.^9,10^

The barriers most commonly reported among the Brazilian adult population are intrapersonal and related to lack of motivation, time and physical limitations.^9^ Overall, these barriers have been identified in specific populations such as students, teachers and police officers,^9^ which may not represent the demographic characteristics, health conditions and lifestyles of the adult populations who use the services available in primary care units.^9,10^

For example, counseling received by patients from professional teams in primary care units may stimulate LTPA.^11,12^ Counseling is a form of orientation based on information, understanding and support that has the aim of facilitating incorporation of new attitudes as behavioral change towards health self-care, such as better dietary habits and smoking cessation, among others.^13–15^ This is an effective and low-cost initiative for promoting LTPA within primary healthcare,^11,12^ and it has also been recognized as a health education action that has the potential to develop individuals’ autonomy to face health-related behavioral change, such as increased active commuting and reduction of sedentary behavior.^16,17^

Although the main barriers to LTPA in the Brazilian population are presented in the literature, few studies have explored them in relation to primary care unit users. Gomes et al.^18^ identified the barriers to LTPA among physically active women in Rio Claro (SP); Silva et al.^19^ assessed the barriers to participation in LTPA programs in Pernambuco; and Häfele and Siqueira evaluated the barriers to behavioral change after physical activity counseling among adults in Pelotas (RS).^20^ From reviewing this literature, it can be seen that there is no evidence with regard to potential individual predictors of barriers in this study population, or whether physical activity counseling could affect the perception of these barriers. Exploring this association may help direct counseling strategies and support management actions for implementing LTPA promotion programs within primary healthcare.

## OBJECTIVE

The aim of this study was to analyze associations between sociodemographic characteristics, health conditions, LTPA levels, physical activity counseling and perceived barriers to LTPA among adult users of primary care units in the Brazilian National Health System.

## METHODS

### Design, study site and ethical considerations

In 2019, a quantitative, observational, cross-sectional study was conducted on a representative sample of adult patients in primary care units (Unidade Básica de Saúde, UBS) in the urban area of São José dos Pinhais, Paraná, southern Brazil. The data used in this study were secondary to the project “Effectivity of community programs for promoting physical activity and reducing sedentary behavior”.^21–23^ This study was reported in accordance with the guidelines for Strengthening the Reporting of Observational Studies in Epidemiology (STROBE).

São José dos Pinhais is a developed city of medium size in the metropolitan region of Curitiba (state capital). The center of this municipality is 18 km from the center of Curitiba. It is situated in an area of 946 km² (79% rural) and has a population of 329,000 inhabitants. Its Human Development (0.758) and Gini (0.459) indices are high. There are 413 healthcare establishments within the city, among which 27 are primary care units (56% in urban area). For this study, data from 15 units in urban areas were selected, since these units are accessible to 90% of the population.

The study was approved by the Research Ethics Committee of the Pontifícia Universidade Católica do Paraná (PUC-PR) (protocol #2.882.260; September 10, 2018) and the procedures used followed the recommendations of the National System for Research Ethics, issued by the National Health Council.

### Sample size

The sample size was estimated from the average number of appointments registered on the website “Transparent Health” during January and February 2019 (N = 34,275). To ensure a representative sample, the proportion of patients receiving PA counseling from healthcare professionals was considered (this proportion was 30%). The confidence level was kept at 95%, the sampling error was 4% and the design effect was 1.5. With these data, the minimum number of participants was estimated as 745. However, to take into account potential losses and refusals, this estimate was increased by 10%, to yield a total of 820 patients. We also agreed to recruit at least 100 extra participants (n = 920) to allow future studies to perform multivariate analyses while avoiding estimation errors. The number of users to be recruited was calculated proportionally to the number of appointments in each primary care unit and ranged from 31 to 92 users.

### Participant selection

Participants were systematically selected based on their position in the waiting room at the primary care unit, counting from one to five, from left to right, using the entrance door as a reference. The third patient was approached. If this person refused to participate or did not meet the inclusion criteria, the first person to their left was selected.

Only adults (≥ 18 years) were invited to participate in the study. Those who did not live in the city or were using the unit for the first time, had any physical limitation that would prevent LTPA (e.g. use of a wheelchair or crutches) or had a cognitive limitation that prevented them from understanding the survey (e.g. hearing loss), were excluded (n = 9).

### Data collection

Ten trained surveyors administered face-to-face interviews between April and October 2019. These took place either before or after a consultation with a healthcare professional and were conducted in a private, reserved room to avoid external influences. The average duration of the interview was 18 minutes (standard deviation, SD = 5 minutes; range = 9-55 minutes).

### Outcome variables: barriers to leisure-time physical activity

The perception of barriers was assessed through an instrument developed for the Brazilian population.^24^ Participants answered the question: *How often do the following affect your LTPA?* Eight individual barriers were evaluated, based on the ones most frequently mentioned in the literature:^9,25^ 1) lack of money; 2) feeling tired; 3) lack of company; 4) lack of time; 5) having an injury or disease; 6) fear of injury; 7) dislike of physical activity or exercise; and 8) feeling too old. A five-point Likert scale was available for each barrier, with the following options: always, often, sometimes, rarely and never.

For analysis purposes, the options “always”, “often” and “sometimes” were grouped and categorized as “yes” (code 1), thus indicating the presence of a barrier. The options “rarely” and “never” were grouped and categorized as “no” (code 0), which consequently represented the lack of a barrier. The total number of barriers was determined as the sum of the eight barriers, ranging from zero to eight.

### Predictor variables

#### Sociodemographic characteristics

The patients’ sex was noted and age was grouped into three categories (young adult: 18-39 years old; middle-aged adult: 40-59 years old; or older adult: ≥ 60 years old). Marital status was classified as single (single/divorced/widowed) or married (married/living with a partner). Skin color was self-reported as one of five categories (white, black, yellow, brown or indigenous) and was categorized as white or nonwhite (all other categories). Education was assessed through five options and was classified into three categories of education: less than elementary school, elementary school or high school or more. Socioeconomic status was assessed using a standard questionnaire^26^ in seven categories (A1 [highest], A2, B1, B2, C, D and E) and was grouped as low-income (C+D+E) or high-income (A+B).

#### Health conditions

The body mass index (BMI) was calculated from self-reported weight and height and was classified into three categories (≤ 24.9 kg/m²; 25.0-29.9 kg/m²; or ≥ 30.0 kg/m²). Presence of chronic disease was assessed through dichotomous responses (no or yes) with regard to medical diagnoses of hypertension, diabetes, dyslipidemia and cardiovascular disease^27^. The number of disease diagnoses was categorized as 0, 1 or ≥ 2. Lastly, the participants were asked to report any continuous use of medications for chronic disease, and this was classified according to the number of prescription medications (0, 1-3 or ≥ 4).

#### Level of leisure-time physical activity

Weekly LTPA in a usual week was measured by means of the leisure module of the long version of the International Physical Activity Questionnaire (IPAQ).^28,29^ The participants reported their weekly frequency and average duration of walking and of moderate and vigorous physical activity. The score for each activity/intensity was obtained as the number of minutes per week (min/week) by multiplying the weekly frequency by the mean daily volume. The total LTPA was obtained by summing the minutes/week of walking + minutes/week of moderate physical activity + (minutes/week of vigorous physical activity*2). Walking and total LTPA were classified in accordance with the World Health Organization guidelines (≥ 150 minutes/week).^30^

#### Physical activity counseling received from healthcare professionals

Physical activity counseling was assessed and recorded based on a dichotomous response (no or yes) to the following question: *During the past year (12 months), in a visit to the healthcare unit, did you receive physical activity counseling while in consultation with a healthcare professional (advice, tips or orientation on physical activity or exercise)?* This measurement had previously been used in similar studies and was adapted to the local context.^13,15,18,20^

#### Data quality control

Quality control was conducted in six steps. First, all research assistants received 20 hours of training on the technical procedures regarding interviews (how to: approach participants, register losses and refusals, administer surveys and code forms), based on an instruction manual that had been developed for the project. The interviewers, who were blinded to the study aims and hypothesis, followed the procedures and were supervised by the field coordinators. Second, a pilot study was conducted using a random sample of 81 participants in three healthcare units to test the procedures for data collection and the understanding of questions in the survey, especially those that had been translated or adapted to the local context. Third, all the participants in the pilot study were surveyed for a second time, after an interval of seven to 10 days, to analyze the temporal stability of the main study variables. The reliability of physical activity counseling was analyzed in terms of the percentage agreement and Cohen's kappa test. The percentage agreement was found to be 88%, and the kappa value was moderate (0.77; P < 0.001). Fourth, the field coordinator was responsible for data entry using the EpiData software (version 3.1, Odense, Denmark). Fifth, data management included an exploratory analysis using the Statistical Package for the Social Sciences (SPSS version 26.0, SPSS Inc., Chicago, Illinois, United States) to identify possible data entry errors and any presence of outliers, and to verify the distribution of all the variables. Lastly, every outlier was checked and corrected when problems were found.

### Statistical analysis

The data were analyzed using descriptive statistics (average, standard deviation and median of the number of barriers) and using the absolute and relative frequency distribution of qualitative variables. The prevalence of each barrier was determined between the categories of predictor variables, and the association was analyzed using chi-square tests (χ^2^) for heterogeneity and linear trend. The normality of the distribution of the number of barriers was analyzed using the Kolmogorov-Smirnov test. The data did not present normal distribution (P < 0.001), and the Mann-Whitney U test was used to compare the number of barriers according to physical activity counseling. Analyses were conducted using the SPSS 26 software, and the significance level was kept at 5%.

## RESULTS

A total of 935 users were approached, with a refusal rate of 14% (n = 134) and loss of 2% (n = 22), thus resulting in a sample of 779 interviewed participants. Most participants were female (69.8%), aged between 18 and 39 years (45.2%), married or living with a partner (64.0%), white (73.0%), with completed high school education or more (50.4%) and of low economic level (71.2%) (Table 1).

**Table 1:** Characteristics of adults assisted within primary healthcare. São José dos Pinhais, Paraná, southern Brazil, 2019 (n = 779)

Variable		Category	n	%
**Sociodemographic characteristics**				
	Sex	Female	544	69.8
		Male	235	30.2
	Age group (years)	18-39	346	45.2
		40-59	283	36.9
		≥ 60	137	17.9
	Marital status	Single	280	36.0
		Married	497	64.0
	Skin color	White	566	73.0
		Nonwhite	209	27.0
	Education level	Less than elementary education	247	31.7
		Elementary education	139	17.8
		High school or more	393	50.4
	Economic level	Low	555	71.2
		High	224	28.8
**Health conditions**				
	Body mass index (kg/m^2^)	< 24.9	248	32.2
		25-29.9	291	37.4
		≥ 30.0	230	29.9
	Hypertension	No	499	64.1
		Yes	280	35.9
	Diabetes	No	657	84.3
		Yes	122	15.7
	Dyslipidemia	No	655	84.1
		Yes	124	15.9
	Coronary artery disease	No	728	93.5
		Yes	51	6.5
	Number of chronic diseases	0	427	54.8
		1	204	26.2
		≥ 2	148	19.0
	Number of prescribed medications	0	387	49.7
		1-3	277	35.6
		≥ 4	115	14.8
**Leisure-time physical activity**				
	Walking (min/week)	< 150	675	86.6
		≥ 150	104	13.4
	Total LTPA (min/week)*	< 150	586	75.2
		≥ 150	193	24.8
	PA counseling received from a healthcare professional	No	444	57.0
		Yes	335	43.0

PA = physical activity; LTPA = leisure-time physical activity;

* minutes/week of walking + minutes/week of moderate PA + (minutes/week of vigorous PA^*^2).

Around 68% were overweight (BMI ≥ 25.0 kg/m²); 35.9% had high blood pressure; 15.7% were diabetic; 15.9% had dyslipidemia; and 6.5% had coronary artery disease. A little over half of the participants reported using medications (50.3%), and 14.8% used four or more different types of pharmacological treatments (Table 1).

Between 13.4% and 24.8% of the participants were active for at least 150 minutes per week, consisting of walking or doing LTPA, respectively. The prevalence of physical activity counseling from healthcare professionals was 43.0% (95% confidence interval, CI: 39.5-46.4%) (Table 1).

**Figure 1:**
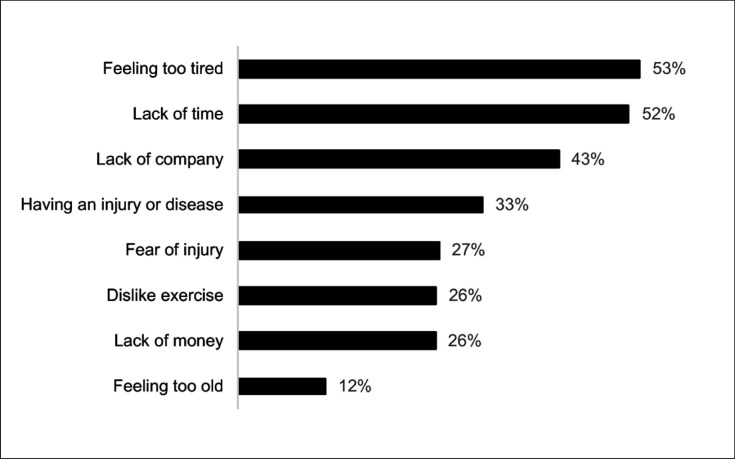
Barriers to leisure-time physical activity reported by adults in primary care units. São José dos Pinhais, Paraná, southern Brazil, 2019 (n = 779).

At least one barrier to LTPA was reported by 89% of the participants. The average number of barriers was 2.70 ± 0.1 (median = 3), and this was similar between the participants who reported receiving physical activity counseling and those who did not (2.73 ± 0.08 versus 2.70 ± 0.10; P = 0.822, respectively). Feeling tired (53%) and lack of time (52%) were the barriers most reported (Figure 1).

**Figure 2:**
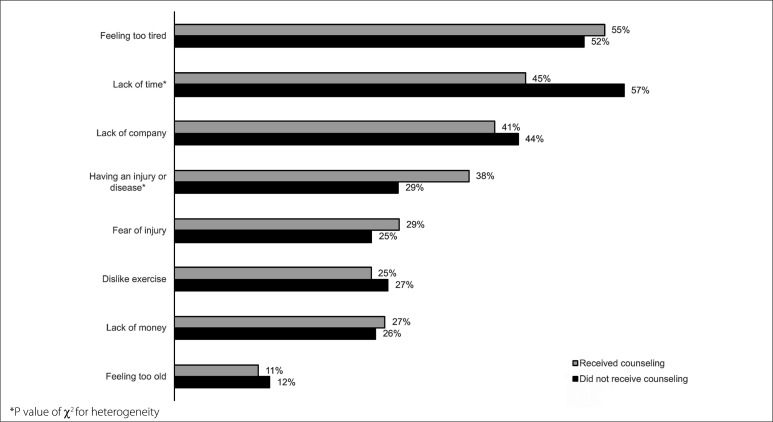
Association between physical activity counseling and perception of barriers reported by adults at primary care units. São José dos Pinhais, Paraná, southern Brazil, 2019 (n = 779).

Physical activity counseling from healthcare professionals was inversely associated with the barrier “lack of time” (45% versus 57%; P < 0.001), but was positively associated with “having an injury or disease” (38% versus 29%; P = 0.008) (Figure 2).

**Figure 3:**
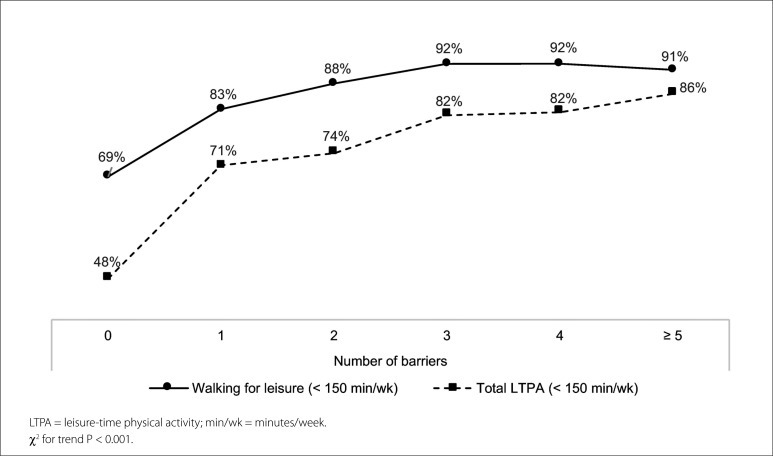
Association between the number of barriers and practicing fewer than 150 minutes per week of walking and total leisure-time physical activity, among adult patients at primary care units. São José dos Pinhais, Paraná, southern Brazil, 2019 (n = 779).

A positive linear trend was observed between the number of barriers and < 150 minutes per week of walking (χ^2^ for trend: 22.3; P < 0.001) and total LTPA (χ^2^ for trend: 38.2; P < 0.001) (Figure 3).

Female sex was associated with the barriers “feeling tired” (56.3% versus 46.8%; P = 0.015), “lack of company” (47.6% versus 31.1%; P < 0.001) and “dislike of exercise” (29.6% versus 18.7%; P = 0.002). Other associations between sociodemographic characteristics, health conditions, LTPA and perception of each barrier are shown in Table 2. Only marital status was not associated with any of the barriers (P > 0.05) (Table 2).

**Table 2:** Association between sociodemographic characteristics, health conditions, leisure-time physical activity and barriers to physical activity reported by adults at primary healthcare units. São José dos Pinhais, Paraná, southern Brazil, 2019 (n = 779)

			Feeling too tired (%)	Lack of time (%)	Lack of company (%)	Having an injury or disease (%)	Fear of injury (%)	Dislike of exercise (%)	Lack of money (%)	Feeling too old (%)
**Sociodemographic characteristics**										
	**Sex (p)**		**0.015** h	0.854^h^	**< 0.001** h	0.699^h^	0.825^h^	**0.002** h	0.129^h^	0.442^h^
		Female	56.3	52.2	47.6	32.9	26.5	29.6	27.8	12.1
		Male	46.8	51.5	31.1	31.5	27.2	18.7	22.6	10.2
	**Age group (years) (p)**		**0.001** t	**< 0.001** t	**0.002** t	**< 0.001** t	**0.003** t	0.119^t^	0.672^t^	0.052^t^
		18-39	58.1	63.0	48.8	22.8	21.1	28.9	24.0	9.5
		40-59	54.1	53.7	38.5	39.6	30.7	24.7	30.4	11.3
		≥ 60	40.1	21.2	35.0	42.3	32.1	22.6	23.4	16.1
	**Marital status (p)**		0.558^h^	0.137^h^	0.510^h^	0.159^h^	0.175^h^	0.385^h^	0.801^h^	0.714^h^
		Single	52.1	48.6	55.7	35.7	29.6	28.2	26.8	12.1
		Married	54.3	54.1	58.1	30.8	25.2	25.4	26.0	11.3
	**Skin color (p)**		0.756^h^	0.117^h^	**0.020** h	0.085^h^	0.318^h^	**0.027** h	**0.031** h	0.662^h^
		White	53.9	53.7	45.2	34.3	25.6	28.4	28.3	11.3
		Nonwhite	52.6	47.4	35.9	27.8	29.2	20.6	20.6	12.4
	**Education level (p)**		0.688^t^	**< 0.001** t	0.228^t^	0.369^t^	0.274^t^	0.181^t^	0.944^t^	**0.033** t
		Less than elementary education	55.1	44.5	38.5	35.6	30.0	23.1	25.9	15.4
		Elementary education	51.1	45.3	46.8	28.8	23.7	27.3	26.6	10.1
		High school or more	53.2	59.0	43.8	31.8	25.7	28.0	26.2	9.7
	**Economic level (p)**		0.227^h^	0.095^h^	0.382^h^	0.704^h^	0.299^h^	**0.049** h	0.082^h^	0.227^h^
		Low	50.0	56.7	40.2	33.5	24.1	21.4	21.9	9.4
		High	54.8	50.1	43.6	32.1	27.7	28.3	27.9	12.4
**Health conditions**										
	**Body mass index (p)**		**0.024** t	0.545^t^	0.602^t^	**0.012** t	0.932^t^	0.778^t^	0.366^t^	0.659^t^
		< 24.9 kg/m^2^	50.0	53.2	41.5	29.0	25.8	27.8	26.2	11.3
		25-29.9 kg/m^2^	51.2	52.6	41.9	30.2	28.2	22.3	22.3	11.0
		≥ 30.0 kg/m^2^	60.4	50.4	43.9	40.0	26.1	29.1	30.0	12.6
	**Hypertension (p)**		0.937^h^	**< 0.001** h	0.268^h^	**< 0.001** h	**0.006** h	0.257^h^	0.257^h^	0.536^h^
		No	53.5	59.1	44.1	27.3	23.4	27.7	24.8	11.0
		Yes	53.2	39.3	40.0	41.8	32.5	23.9	28.6	12.5
	**Diabetes (p)**		0.080^h^	**< 0.001** h	0.841^h^	**0.002** h	**0.006** h	0.273^h^	**0.042** h	0.069^h^
		No	52.1	54.8	42.5	30.3	24.8	25.6	24.8	10.7
		Yes	60.7	36.9	43.4	44.3	36.9	30.3	33.6	16.4
	**Dyslipidemia (p)**		0.878^h^	**0.015** h	0.867^h^	**< 0.001** h	**0.008** h	0.598^h^	0.146^h^	0.152^h^
		No	53.3	53.9	42.7	29.6	24.9	26.0	25.2	10.8
		Yes	54.0	41.9	41.9	47.6	36.3	28.2	31.5	15.3
	**Coronary artery disease (p)**		0.166^h^	0.191^h^	0.611^h^	**< 0.001** h	0.435^h^	0.239^h^	0.230^h^	**0.006** h
		No	52.7	52.6	42.9	30.9	26.4	25.8	25.7	10.7
		Yes	62.7	43.1	39.2	54.9	31.4	33.3	33.3	23.5
	**Number of chronic diseases (p)**		0.646^t^	**< 0.001** t	0.399^t^	**< 0.001** t	**0.002** t	0.965^t^	0.142^t^	0.185^t^
		0	53.2	59.5	45.4	24.4	23.4	26.9	24.8	11.0
		1	52.0	47.5	35.3	39.2	25.5	23.5	25.0	9.3
		≥ 2	56.1	36.5	44.6	46.6	37.8	28.4	31.8	16.2
	**Number of prescribed medications (p)**		0.673^t^	**< 0.001** t	0.479^t^	**< 0.001** t	**< 0.001** t	0.700^t^	**0.040** t	**0.037** t
		0	53.7	62.0	43.9	22.2	22.0	27.4	23.0	9.6
		1-3	50.9	48.7	41.5	36.8	27.8	24.5	28.5	12.3
		≥ 4	58.3	26.1	40.9	56.5	40.0	27.0	31.3	16.5
**Leisure-time physical activity (LTPA)**										
	**Walking (min/wk) (p)**		**0.015** h	**< 0.001** h	**0.001** h	0.861^h^	0.510^h^	**0.003** h	0.083^h^	0.738^h^
		< 150	55.1	55.4	45.0	32.6	27.1	28.1	27.3	11.7
		≥ 150	42.3	29.8	26.9	31.7	24.0	14.4	19.2	10.6
	**Total LTPA (min/wk)** * **(p)**		**< 0.001** h	**< 0.001** h	**0.001** h	0.314^h^	0.508^h^	**< 0.001** h	0.107^h^	0.102^h^
		< 150	57.2	58.0	46.9	33.4	27.3	29.7	27.6	12.6
		≥ 150	42.0	33.7	29.5	29.5	24.9	16.1	21.8	8.3

* min/wk = minutes/week of walking + minutes/week of moderate physical activity (PA) + (minutes/week of vigorous PA^*^2);

t χ^2^ for linear trend;

h χ^2^ for heterogeneity.

## DISCUSSION

In this study, we sought to explore intrapersonal correlates as potential predictors of LTPA barriers perceived by adult patients in primary care units in a city in southern Brazil. The quantitative approach adopted allowed exploration of associations between sociodemographic characteristics, health conditions, LTPA, physical activity counseling and the prevalence of each barrier in a representative sample of adults. These were positive and innovative aspects of this study. The analyses among users of primary care units assisted in enabling understanding of how physical activity counseling from healthcare professionals may have an impact on the barriers associated with physical inactivity in this population. Moreover, the variables were measured through valid, internationally standardized protocols and instruments, which allowed comparisons with similar studies.

This study presents a sample with an important sociodemographic characteristic, representing a population that is more vulnerable to physical inactivity in Brazil (e.g. women, low-income individuals, with chronic diseases and who depend on continuous medication). This is relevant since community programs to promote physical activity in Brazil allow free access for the population with these characteristics.^23,31^

In this study, “feeling too tired” and “lack of time” were the most prevalent barriers, and this agreed with the results from other studies.^9,20,25,32^ These reports may be partially explained by the participants’ daily routines (work, commuting and household activities), which would impact their liveliness and time available for LTPA. Therefore, orientations and counseling actions from healthcare professionals could include all four domains of physical activity (leisure, transportation, occupation and household), with the aim of raising awareness about the need to reduce sedentary time.^9,30^

Intrapersonal, interpersonal and environmental barriers faced by adolescents, adults and older adults have been widely reported and identified in the literature.^9,19,32^ However, these reports cannot be extrapolated for the general population. In fact, among adult patients seen at primary care units, the main barriers are health conditions and lack of appropriate locations for exercising near their homes, among others.^18,19^ However, these barriers differ among individuals. For example, a healthy young single male who is university-educated and has a high income, and who plays sports regularly and intends to swim in a gym, will probably have barriers that are different to those of a middle-aged black female who is a day laborer with a low income and is physically inactive and hypertensive, and who intends or needs to participate in guided walking groups offered at the primary care unit near home.^18,19^

Physical activity counseling was inversely associated with the “lack of time” barrier but positively associated with “having an injury or disease”. The time available for activities is a multifactorial issue that may not be easily changed.^9,32^ However, counseling is a strategy for health promotion that involves orientation and support from professionals. This action may contribute to a more positive perception among individuals with regard to organizing their time for LTPA. In this manner, the relevance of counseling in the process of behavioral change is strengthened.^11–13^ The positive association between counseling and the barrier of “having an injury or disease” may be explained by studies that have shown that there is a higher probability that older adults and those with chronic disease are the ones receiving counseling.^20,33^

The number of barriers was associated with the prevalence of < 150 minutes per week of walking and total LTPA. Similar results were found in a population study in Pelotas (RS), another mid-sized city in the southern region of Brazil.^25^ These results are essential and reiterate the idea that counseling needs to be directed at the individual level to stimulate LTPA and reduce the perception of barriers. Therefore, it is clear that reducing barriers should be at the center of healthcare professionals’ actions towards increasing the level of LTPA in this population.

Multiple sociodemographic characteristics and health conditions presented associations with barriers to LTPA in the expected ways. For example, the perceptions of “feeling too tired”, “lack of company” and “dislike of exercise” were also found to be more likely among women in other studies. These perceptions can be explained by double shifts, lack of support from a partner and, consequently, low motivation for activities.^9,19,25,32^ Also, for example, older adults (with physical limitations, chronic disease, arthritis or arthrosis) may fear that higher-intensity LTPA could result in pain or injuries.^9,19,25,32^ These results highlight the need for programs to promote LTPA in the Brazilian National Health System and the need to identify barriers to participation in the activities that are offered. Thus, healthcare teams should aim to create strategies to reduce the impact of barriers, so that program users may be more physically active.

The results from this study provide important information that needs to be considered in planning, implementing, conducting and maintaining actions integrated with counseling from healthcare professionals, to reduce barriers to LTPA. Furthermore, these results can help leverage community programs for physical activity promotion within primary care, given that such programs can affect the population's interest in, search for and involvement in these activities.^18,19,32,34^

Programs need to be developed and directed according to the barriers perceived by different groups stratified in terms of sex, age, chronic disease and LTPA levels. For example, health-care professionals may stimulate and counsel users to seek community programs for LTPA that are available in the city, based on these perceptions of barriers. In turn, the programs need to offer pleasant group-based activities that can be performed in public open spaces and which are diversified in time, type and intensity so as to engage the population.^6,8,18,19,32,34,35^

Continuous and adequate physical activity counseling could be emphasized for people with chronic disease or fear of illness, and for people who are insufficiently active during their leisure time.^11,22^ This could make it easier for primary care unit users to understand that regular physical activity is not a potential cause of injury or pain.^9^ The Physical Activity Guidelines for the Brazilian Population can contribute to a better understanding of physical activity among the population and help healthcare professionals regarding the content for counseling.^36^ Also, healthcare professionals could encourage users to attend public open spaces (e.g. parks, squares, sports and leisure centers and fitness zones) that are easily accessible and free, for LTPA.^19,31,32^

Some limitations of this study need to be considered in order to adequately interpret and extrapolate these results. First, the sample was limited to adult patients at primary care units in the urban area of a mid-sized city in southern Brazil. Second, these units did not have a physical education professional as part of their healthcare teams, which may have affected the perception of barriers. Third, the quantitative approach using a short, standardized survey to evaluate intrapersonal barriers did not allow much depth when exploring interpersonal or environmental matters of relevance to implementation of physical activity promotion programs in the community. Therefore, it was impossible to capture contextual information that could explain or signify feelings towards barriers. This would only have been possible through a qualitative or mixed-method approach.^10^ Lastly, the cross-sectional design limited the capacity to establish causality between predictor variables and the outcomes.

## CONCLUSION

The perceived barriers to physical activity that were most often reported were “feeling too tired” and “lack of time”. These barriers differed in importance according to sociodemographic characteristics, health conditions and levels of LTPA. Physical inactivity increased with increasing numbers of perceived barriers.

Future studies may advance the analyses and explore other interpersonal and environmental aspects of barriers through using mixed methods among patients at primary care units in larger cities or rural areas of smaller municipalities. It is also relevant to assess the effectiveness of counseling actions provided by trained professionals, using an evidence-based technical protocol to reduce the perception of barriers against LTPA.
